# Age-Related Variations in Sleep Duration and Quality Before, During, and After Stroke Among Hospitalised Stroke Patients

**DOI:** 10.7759/cureus.94623

**Published:** 2025-10-15

**Authors:** Daniel E Ekwueme, Stephanie Dahr, Siddharth Musunuri, Sonakshi Nemchand, Thivyaa A Ravindran, Thoufeer Ali, Akhila Haroon, Kulsum Laaeba Faiz, Omneya T Abumiddain, Mujtaba M Mohammed, Mohammed Labeeb Iqbal, Shatakshi -, Ling Fung Chan, Kenvael G Le Cam, Abdullah B Ashraf, Maliha Khabir, Sameer Almashta

**Affiliations:** 1 Internal Medicine, Wrexham Maelor Hospital, Wrexham, GBR; 2 Stroke, Wrexham Maelor Hospital, Wrexham, GBR; 3 General Internal Medicine, Wrexham Maelor Hospital, Wrexham, GBR

**Keywords:** age differences, hospitalisation, post-stroke care, rehabilitation, sleep duration, sleep quality, stroke

## Abstract

Introduction

Stroke is a leading cause of mortality and long-term disability worldwide, with sleep disturbance increasingly recognised as both a risk factor and a determinant of recovery. Despite its clinical importance, sleep is seldom incorporated into routine stroke care. This study investigated age-related variations in sleep duration and quality before stroke, during hospitalisation, and after discharge.

Methods

A retrospective cohort study was conducted at Wrexham Maelor Hospital, North Wales, including 224 stroke patients admitted between March 2024 and March 2025. Stroke admissions were identified from the Sentinel Stroke National Audit Programme, with additional demographic and clinical data retrieved from electronic records. Sleep characteristics were assessed via structured questionnaires covering pre-stroke, admission, and post-discharge phases. Outcomes included average sleep duration (hours) and subjective quality (4-point scale), which were analysed using repeated measures ANOVA.

Results

Across all age groups, sleep duration and quality declined significantly during hospitalisation and showed partial recovery post-discharge. Pre-stroke sleep duration averaged approximately seven hours, dropping to 6.1 hours in younger patients, 6.4 hours in middle-aged patients, and 5.7 hours in older adults during admission. Sleep quality fell from ~3.0 pre-stroke to 2.1-2.5 during admission. Post-discharge, both measures improved but did not fully return to pre-stroke levels. Between-group ANOVAs revealed no statistically robust age-related differences in sleep duration, as p-values were consistently greater than 0.05 across groups. The only exception was observed in the 18-49 age group for pre-stroke sleep duration, where a statistically significant difference emerged. However, adjusted effect size estimates (ε² and ω²) were consistently near zero, indicating that the apparent significance may be inflated. These corrections suggest that the observed difference is unlikely to reflect a meaningful or clinically important effect, despite the large raw η² values reported.

Conclusion

Hospitalisation for stroke was associated with marked reductions in both sleep duration and quality across all age groups, with only partial recovery observed following discharge. However, these differences did not reach statistical significance. These findings underscore the importance of incorporating systematic evaluation and targeted interventions for sleep disturbances into comprehensive stroke care pathways, in order to optimise recovery and long-term patient outcomes.

## Introduction

Stroke is a major neurological emergency that has both direct and indirect health, social, and economic costs. Stroke is defined as an acute brain injury caused by vascular occlusion or intracerebral haemorrhage, resulting in brain ischaemia, neuronal injury, and subsequent long-term disability or death [1,2]. Stroke is globally one of the leading causes of mortality and chronic disability in the adult population, with the ageing population amplifying the burden [3]. While the use of advanced interventions, such as thrombolysis and thrombectomy, can improve short-term outcomes, stroke survivors often struggle with long-term recovery, influenced by many interrelated factors, including comorbidities and psychosocial determinants [4-6]. Although rehabilitation is a crucial part of recovery, it is just one component of the broader continuum of care after stroke [7].

Within the variety of factors influencing recovery, sleep is a significant yet frequently overlooked domain. Sleep is a complex biological state regulated by both circadian rhythms and homeostatic mechanisms. It has been shown to be critical for cognitive function, emotional regulation, and physiological repair [8,9]. There is growing evidence that poor sleep, whether presenting as insomnia, excessive sleepiness, or general disturbance, may impact not only recovery trajectories but may also contribute to stroke occurrence via mechanisms such as hypertension, systemic inflammation, and endothelial dysfunction [10,11]. There is also evidence that many stroke patients experience disrupted sleep during hospitalisation and ongoing sleep difficulties after discharge, which in turn may affect the likelihood of them being willing or able to access timely rehabilitation and follow-up care [12,13]. Nevertheless, sleep is seldom examined or incorporated into routine assessment in stroke care pathways.

This study aims to examine age-related variations in sleep duration and quality before stroke onset, during hospital admission, and following discharge among hospitalised stroke patients. Through an age-stratified analysis, it seeks to identify patterns of sleep disturbance across age cohorts and to assess their potential implications for stroke recovery. The findings are intended to guide the development of age-appropriate, patient-centred approaches to post-stroke care.

## Materials and methods

Study design

This retrospective cohort study was conducted at Wrexham Maelor Hospital, a regional stroke unit in North Wales, using data collected between March 2024 and March 2025. A total of 224 people were included in this study.

Data sources

Stroke admissions were identified through the Sentinel Stroke National Audit Programme (SSNAP), a national registry that sets standards for stroke care data across the United Kingdom. Once stroke cases were identified, additional demographic, clinical, and diagnostic data were retrieved from the Welsh Clinical Portal. These included stroke classification (type and subtype), comorbidities, and any documented sleep-related observations.

Data collection and sleep assessment

The questionnaire was administered retrospectively following discharge and collected clinical variables, including patient demographics, stroke type (ischaemic or haemorrhagic), anatomical location, and subtype. Sleep characteristics were assessed at the following time points: pre-stroke, during hospitalisation, and post-discharge. Participants completed a number of questions related to average sleep duration, perceived quality of sleep (on a 4-point scale), and whether they experienced sleep disruption. During hospitalisation, specific questions were asked about the nature of sleep disturbances, length of stay, ward location, whether concerns were raised with healthcare professionals, and if any interventions were initiated. After discharge, questions focused on sleep variables and whether patients discussed their ongoing sleep problems with their general practitioner or hospital consultant.

Questionnaire provenance and permissions

Sleep quality was measured using an investigator-developed 4-point Likert-type item. The exact item wording and response options are reproduced in Appendix A. Because this instrument was developed by the study team and not adapted from a copyrighted source, no third-party permissions were required.

Age stratification

Participants were categorised into three age groups for comparative analysis: 18-49 years, 50-74 years, and ≥75 years. This stratification aimed to reflect key differences in stroke risk profiles, recovery potential, and broader health and social care needs, allowing for a focused evaluation of sleep outcomes across age cohorts.

Eligibility criteria

Eligible participants were adults aged ≥18 years with a confirmed stroke diagnosis and available clinical documentation, as well as completed questionnaire data on sleep. Patients were excluded if they had a diagnosis of transient ischaemic attack (TIA), had incomplete clinical records, or were unable to provide informed consent or complete the questionnaire due to severe cognitive or communicative impairment.

Sample size and power

Although no prior sample size calculation was performed, the final cohort of 224 patients was considered sufficient to allow subgroup comparisons by age category (18-49, 50-74, ≥75 years) and across the three phases of interest (pre-stroke, hospitalisation, post-discharge). Post-hoc power analyses were conducted using paired within-subject comparisons (pre-stroke vs admission, admission vs post-stroke). To detect a one-hour change in sleep duration with 80% power at a two-sided α = 0.05, approximately 70 participants would be required, whereas detection of a smaller 0.5-hour change would require around 280 participants. For sleep quality (4-point scale), a 0.5-point difference would require approximately 50 participants, and a 0.3-point change would require around 130 participants. With 224 patients, the study was more than adequately powered.

Statistical analysis

All analyses were conducted using repeated measures designs to account for within-subject changes across the three phases (pre-stroke, admission, post-stroke). Continuous outcomes, including average hours of sleep, were analysed using repeated measures analysis of variance (ANOVA) with phase as the within-subject factor. Subjective sleep quality, assessed on a 4-point scale, was treated as a continuous variable for parametric testing and evaluated within the same ANOVA framework. Statistical analyses were performed using Statistical Product and Service Solutions (SPSS, version 31; IBM SPSS Statistics for Windows, Armonk, NY). A p-value of <0.05 was considered statistically significant.

## Results

The study was more than adequately powered with a cohort of N = 224 (minimal detectable differences ≈0.56 hours for duration and ≈0.23 points for quality at α=0.05), distributed as 18-49: N = 10 (4.5%); 50-74: N = 104 (46.4%); and ≥75: N = 110 (49.1%). The observed in-hospital changes - about 1.0 hour less sleep and a 0.5-point drop in quality - exceeded these thresholds, implying very high achieved power (>99%).

Across all age groups, sleep duration (hours, mean ± SD) and sleep quality (1-4 scale, mean ± SD) declined during hospitalisation and partially recovered after discharge (Table 1). Pre-stroke duration averaged ~7 hours/night across age categories (18-49: 7.00 ± 1.75; 50-74: 7.10 ± 1.69; ≥75: 6.96 ± 1.66). During admission, duration decreased across all groups, most notably in ≥75 (5.72 ± 3.23 h), with reductions also in younger (6.10 ± 3.20 hours) and middle-aged patients (6.40 ± 2.98 hours). Post-discharge, duration improved to near or slightly above pre-stroke values (6.82-7.30 hours). Sleep quality followed the same pattern: pre-stroke means ~3.0 declined during admission (18-49: 2.10 ± 1.05; 50-74: 2.48 ± 1.02; ≥75: 2.46 ± 1.01) with partial recovery (2.82-3.04) that did not fully return to baseline. Collectively, these data indicate a pronounced adverse effect of hospitalisation on both duration and quality of sleep across age strata, with only partial resolution after discharge (see Tables 2-3 for inferential statistics; two-tailed, α = 0.05).

**Table 1:** Average sleep duration and quality across age groups Values are mean ± SD (duration in hours; quality 1–4, higher = better). Age-group sample sizes (N = 224): 18–49: N = 10 (4.5%); 50–74: N = 104 (46.4%); ≥75: N = 110 (49.1%). Denominators for percentages use the analysis N unless specified.

Age range	(Mean) Hours pre-stroke ± SD	(Mean) Quality pre-stroke ± SD	(Mean) Hours during admission ± SD	(Mean) Quality during admission ± SD	(Mean) Hours post-stroke ± SD	(Mean) Quality post-stroke ± SD
18-49	7.00 ± 1.75	3.00 ± 0.89	6.10 ± 3.20	2.10 ± 1.05	7.30 ± 2.06	2.90 ± 0.99
50-74	7.10 ± 1.69	2.95 ± 0.86	6.40 ± 2.98	2.48 ± 1.02	7.24 ± 2.12	3.04 ± 0.95
75+	6.96 ± 1.66	3.04 ± 0.80	5.72 ± 3.23	2.46 ± 1.01	6.82 ± 2.24	2.82 ± 0.92

**Table 2:** Summary results of ANOVA for sleep duration across age groups (18–49, 50–74, ≥75 years) and phases (pre-stroke, admission, post-stroke) and results of the statistical analysis. Data shown: F(df₁, df₂), p (two-tailed), and η² (effect size). Analyses were run between each age stratum (18–49, 50–74, ≥75) across phases (pre-stroke, admission, post-stroke). Significance threshold: α = 0.05 (significant if p < 0.05).

Age group	Phase	Hours, F (df)	p-value	η² (raw)
18–49	Pre-stroke	F(6,3) = 17.50	0.020	0.97
18–49	Admission	F(6,3) = 2.26	0.269	0.82
18–49	Post-stroke	F(6,3) = 3.52	0.165	0.88
50–74	Pre-stroke	F(23,80) = 0.64	0.886	0.15
50–74	Admission	F(23,79) = 0.61	0.912	0.15
50–74	Post-stroke	F(23,80) = 1.22	0.250	0.26
75+	Pre-stroke	F(23,86) = 1.35	0.160	0.27
75+	Admission	F(23,86) = 0.53	0.959	0.12
75+	Post-stroke	F(23,86) = 1.27	0.214	0.25

**Table 3:** Summary results of ANOVA for sleep quality across age groups (18–49, 50–74, ≥75 years) and phases (pre-stroke, admission, post-stroke) and results of the statistical analysis Data shown: F(df₁, df₂), p (two-tailed), and η² (effect size). Analyses were run between each age stratum (18–49, 50–74, ≥75) across phases (pre-stroke, admission, post-stroke). Significance threshold: α = 0.05 (significant if p < 0.05).

Age group	Phase	Quality, F (df)	p-value	η² (raw)
18–49	Pre-stroke	–	–	–
18–49	Admission	F(6,3) = 0.95	0.564	0.66
18–49	Post-stroke	–	–	–
50–74	Pre-stroke	F(23,80) = 0.81	0.715	0.14
50–74	Admission	F(23,79) = 0.55	0.948	0.14
50–74	Post-stroke	F(23,80) = 1.10	0.369	0.24
75+	Pre-stroke	F(23,86) = 1.44	0.117	0.28
75+	Admission	F(23,86) = 1.12	0.340	0.23
75+	Post-stroke	F(23,86) = 1.25	0.227	0.25

Between-group analyses of sleep outcomes

As shown in Table 2, between-group ANOVAs were conducted within each age stratum (18-49, 50-74, ≥75 years) across pre-stroke, admission, and post-stroke phases. 

Phase effects (duration): As represented in Table 2, in the 18-49 group, a significant pre-stroke effect was observed (F(6,3) = 17.50, p = 0.020, η² = 0.97), though this was likely inflated by very small degrees of freedom (df = 3). No significant differences emerged during admission or post-stroke (all p > 0.16). In both the 50-74 and ≥75 groups, no significant effects were found at any time point (all p > 0.16-0.95). Although some raw η² values appeared small to moderate, corrected effect sizes (ε², ω²) were near zero, indicating negligible explanatory value.

Phase effects (quality): As illustrated in Table 3, for 18-49 patients, pre-stroke and post-stroke ANOVAs could not be computed due to zero variance; the admission model was non-significant (F(6, 3) = 0.95, p = 0.564). In both the 50-74 and ≥75 groups, no significant effects were observed at any phase (all p > 0.11-0.95). Although raw η² values occasionally indicated moderate variance explained, corrected indices confirmed negligible group-level effects. Across all age groups, between-group ANOVAs did not reveal robust or clinically meaningful differences in sleep duration or quality. Moderate-to-large raw η² values were artefacts of small or unbalanced subgroup sizes, with corrected effect size estimates consistently near zero.

## Discussion

This research examined age-related patterns in sleep duration and quality among stroke patients across three phases: before stroke, during hospitalisation, and after discharge. The principal finding was that hospitalisation was consistently associated with a significant reduction in both sleep duration and quality, with partial but incomplete recovery after discharge. Importantly, between-group ANOVAs did not identify a statistical difference between age strata, indicating that the impact of stroke and hospitalisation on sleep is a largely universal phenomenon across the adult age spectrum.

Our results are consistent with existing literature that highlights the disruption of sleep during acute hospital admission for stroke. Previous studies have shown that stroke patients often experience reduced sleep duration during hospitalisation, with nocturnal disturbances attributed to environmental factors, medical interventions, and psychological stress [12,13]. The observed reduction of approximately one hour of sleep and a 0.5-point decline in subjective quality align closely with earlier prospective cohorts, where reductions of 45-90 minutes of sleep and significant deterioration in subjective sleep quality were reported during hospital stays [14-16]. Our post-hoc power analysis confirmed that these changes were well above the minimal detectable thresholds, with achieved power estimates >99%, lending strong statistical confidence to these observations.

Interestingly, the present study did not demonstrate significant age-related differences in either sleep duration or quality, despite some raw effect sizes suggesting small-to-moderate variance explained. Adjusted indices (ε² and ω²) consistently approached zero, indicating that any apparent group-level variation was artefactual. This finding contrasts with prior reports suggesting that older stroke survivors are more vulnerable to disrupted sleep, owing to comorbidities, polypharmacy, and frailty [14,15]. However, it supports other studies that found hospital-related factors, such as noise, light exposure, and interruptions for monitoring, to be stronger predictors of poor sleep than patient demographics [16]. Our results, therefore, add to the emerging consensus that hospital environmental and procedural factors outweigh demographic determinants in shaping sleep outcomes after stroke.

Partial recovery of both sleep duration and quality after discharge observed in our cohort is also consistent with previous follow-up studies, which noted improvements in sleep parameters over the first three to six months post-stroke [17]. However, our data demonstrate that post-stroke sleep quality remained below pre-stroke levels across all age groups, suggesting persistent deficits. This echoes findings from large registry-based studies, where up to 40% of patients reported ongoing insomnia or poor sleep quality months after discharge [13]. Persistent disturbances may reflect the bidirectional relationship between stroke and sleep, in which pre-existing sleep pathology predisposes to stroke, while the neurological insult further disrupts sleep-wake regulation [10,11].

Taken together, these findings highlight the need for systematic assessment and management of sleep in stroke care pathways. Although rehabilitation services traditionally prioritise motor and cognitive outcomes, our results suggest that sleep is an important but under-recognised determinant of recovery. Previous interventional studies have shown that non-pharmacological approaches, including sleep hygiene strategies and environmental modifications (e.g., noise and light reduction in wards), can improve sleep quality and potentially enhance recovery trajectories [18]. The present study reinforces these findings and suggests that such strategies should be considered across all age groups, rather than targeted solely at older patients.

Strengths of the study

The study has several notable strengths. First, the relatively large sample size (N = 224) provided sufficient statistical power to detect meaningful differences in sleep duration and quality across phases of care, ensuring robust within-subject comparisons. Second, the use of a national audit registry (e.g., SSNAP) in combination with electronic clinical records strengthened the accuracy of stroke case identification and minimised the risk of diagnostic misclassification. Third, the inclusion of age-stratified analyses (18-49, 50-74, and ≥75 years) allowed for the exploration of demographic patterns, enhancing the relevance of findings across the adult life course. Finally, by assessing sleep outcomes at three clinically important time points, namely, pre-stroke, during hospitalisation, and after discharge, this study provides a comprehensive overview of sleep changes across the continuum of stroke care, highlighting hospitalisation as a critical period of vulnerability.

Limitations

Several limitations should be acknowledged. First, the retrospective design and reliance on patient self-report for sleep assessment introduce the potential for recall bias and subjective variability. Objective measures, such as the Epworth scale of sleepiness or polygraphy, were not used and could have provided more precise insights into sleep architecture and circadian rhythm disturbances. Second, the study was conducted in a single regional stroke centre, which may limit the generalisability of findings to other populations or healthcare systems with different ward environments, staffing patterns, or rehabilitation practices. Third, while subgroup analyses by age were undertaken, sample sizes within certain strata (particularly the younger group) were small, leading to unstable variance estimates and inflated effect sizes, as reflected in the statistical output. Additionally, we did not account for potentially confounding factors such as stroke severity, lesion location, comorbid sleep disorders (e.g., sleep apnoea), or medication use, all of which may influence sleep patterns.

Future directions

Future studies should adopt prospective, multicentre designs with larger and more balanced age cohorts to validate these findings and improve external validity. The use of objective sleep-monitoring techniques would enhance the accuracy of sleep characterisation and allow for the exploration of mechanistic links between neurological injury and sleep disruption. Furthermore, research should examine the role of modifiable environmental and procedural factors within stroke units, such as noise, light, and frequency of nocturnal observations, which may disproportionately contribute to in-hospital sleep disturbance. Finally, interventional trials evaluating pharmacological and non-pharmacological strategies to improve sleep in stroke survivors are warranted, with a focus on understanding whether improved sleep translates into enhanced rehabilitation outcomes, reduced recurrence risk, and better long-term quality of life.

## Conclusions

This study provides evidence that sleep is markedly disrupted during hospitalisation for stroke, with both duration and quality significantly reduced across all age groups and only partially recovering after discharge. Importantly, age-related differences were minimal, suggesting that hospital-related environmental and procedural factors, rather than demographic characteristics, are the primary drivers of post-stroke sleep disturbance. These findings are consistent with and extend previous research, reinforcing the view that sleep should be recognised as a critical but under-addressed component of stroke care. Integrating systematic sleep assessment and targeted interventions into routine clinical pathways may represent a valuable opportunity to enhance rehabilitation, improve quality of life, and reduce long-term morbidity among stroke survivors.

## Appendices

**Table 4:** Structured questionnaire used in the stroke sleep study Stroke Sleep Questionnaire (author-developed). This self-completed English (UK) instrument (telephone/online; ~3-5 minutes) captures sleep duration, perceived sleep quality, and sleep disruption across three recall windows: pre-stroke, during hospitalization, and post-discharge. Permissions: investigator-developed instrument; no third-party permissions required. The authoritative wording, response options, recall windows, and scoring are provided.

Consent:
Patient's Initials:
Patient's CRN:
Age:
Gender:
Type of Stroke-(Haemorhagic/Ischaemic):
Location/Side of Stroke-(Right or Left):
Area-(Anterior/Posterior):
Kind of Stroke-(LACS, PACS, TACS, POCS):
How was your sleep before admission?:
How many hours? (Sleep duration (hours) - “How many hours did you usually sleep?” response __ . __ hours (0.0-24.0; 0.5-h increments):
Quality- (Sleep quality (4-point Likert) - “Overall, how would you rate your sleep quality?” 1 = Very poor; 2 = Poor; 3 = Good; 4 = Very good):
Interrupted?-“Did you experience sleep disruption (trouble falling asleep, staying asleep, or waking too early)”(Yes/No):
How was your sleep during admission?:
How many hours?(Sleep duration (hours) - “How many hours did you usually sleep?” response __ . __ hours (0.0-24.0; 0.5-h increments):
Quality-(Sleep quality (4-point Likert) - “Overall, how would you rate your sleep quality?” responses 1 = Very poor; 2 = Poor; 3 = Good; 4 = Very good):
Cause of worsening sleep - I.Disability II. Pain III. Anxiety/Stress IV. Lighting V. Noise VI. Intervention(Vitals, bloods etc):
Duration of stay in the hospital (Days):
Ward:
How was your sleep after discharge?:
How many hours? (Sleep duration (hours) - “How many hours did you usually sleep?” response __ . __ hours (0.0-24.0; 0.5-h increments):
Quality: (Sleep quality (4-point Likert) - “Overall, how would you rate your sleep quality?” responses 1 = Very poor; 2 = Poor; 3 = Good; 4 = Very good):
Interrupted?-“Did you experience sleep disruption (trouble falling asleep, staying asleep, or waking too early)”(Yes/No):
Place of discharge-(Home, Nursing Home, Residential Home, Rehabilitation Hospital, Tertiary Hospital ):
Did you speak to your GP?-(yes/no):
Did you speak to a medical consultant?-(yes/no):
